# Sensitive Skin Syndrome: A Low-Noise Small-Fiber Neuropathy Related to Environmental Factors?

**DOI:** 10.3389/fpain.2022.853491

**Published:** 2022-03-25

**Authors:** Laurent Misery, Adeline Bataille, Matthieu Talagas, Christelle Le Gall-Ianotto, Maxime Fouchard, Flavien Huet, Anne-Sophie Ficheux, Alain-Claude Roudot, Joachim W. Fluhr, Emilie Brenaut

**Affiliations:** ^1^Univ Brest, LIEN, Brest, France; ^2^Department of Dermatology, Venereologie and Allergology, Charité Universitaetsmedizin, Berlin, Germany

**Keywords:** sensitive skin, small-fiber neuropathy, itch, pain, environment

## Abstract

**Background and Objectives:**

Patients frequently complain of mild, transient, unpleasant skin sensations that cannot be diagnosed as common neuropathies. Dermatologists have termed these symptoms “sensitive skin syndrome.” This narrative review was performed for a better knowledge by other specialists.

**Databases and Data Treatment:**

Publications on pain in sensitive skin syndrome were obtained from PubMed.

**Results:**

There is a growing body of data supporting the concept that sensitive skin is a type of small-fiber neuropathy. The arguments are based on clinical data, a decrease in intra-epidermal nerve fiber density, quantitative sensory testing abnormalities and an association with irritable bowel syndrome and sensitive eyes. Sensitive skin is triggered by environmental factors. Sensitive skin is a frequent condition, with a lifetime prevalence of ~50% according to self-reports.

**Conclusions:**

Mild levels of skin pain or itch are frequently experienced by patients, who rarely report them. There is a need for a better knowledge of sensitive skin because it can be the first level of small-fiber neuropathies.

## Introduction

Patients frequently experience mild and transient itch, skin pain or other unpleasant skin sensations that cannot be diagnosed as common neuropathies. Consequently, these conditions are frequently considered functional disorders without any precise diagnosis. Neuropathic itch may have numerous causes (1). The most frequent etiology of neuropathic itch in the skin is sensitive skin syndrome (1, 2). Although first reported after World War II (3), sensitive skin became an area of interest in mainly from the 1980s (4, 5). Sensitive skin was reported as a syndrome in 2006 (6).

The International Forum for the Study of Itch (IFSI), which is the counterpart of the International Association of the Study of Pain (IASP), initiated a special interest group on sensitive skin and provided a consensus definition (7). Using the Delphi method, this group defined sensitive skin as “A syndrome defined by the occurrence of unpleasant sensations (stinging, burning, pain, pruritus, and tingling sensations) in response to stimuli that normally should not provoke such sensations. These unpleasant sensations cannot be explained by lesions attributable to any skin disease. The skin can appear normal or be accompanied by erythema. Sensitive skin can affect all body locations, especially the face” (7). Some subclassifications were proposed (8). Among them, one's suggested to oppose primary sensitive skin (without any underlying skin disease) to secondary sensitive skin (with an underlying skin disease) (8).

Until now, non-dermatologists are not familiar with this condition. The aim of this narrative study was to provide interesting and useful data for the pain management community.

## Clinical Presentation

Subjects with sensitive skin usually have minimally visible skin lesions or no lesions at all, but erythema is often observed. These symptoms may occur spontaneously, but they are most frequently induced by exogenous triggers such as cosmetics, environmental conditions [e.g., ultraviolet (UV) light, temperature, or wind] and psychological (e.g., stress) and hormonal factors (e.g., the menstrual cycle) (6, 9, 10). In most patients, symptoms occur within 1 h following exposure to trigger factors and may persist for minutes or even hours (11). There is no proven relationship between sensitive skin and psychological symptoms (12).

Sensitive skin must be distinguished from irritated skin and sensitized skin (Table 1). Irritated skin presents the same clinical symptoms but occurs in response to factors that might be irritating to the general population (e.g., shaving, laser treatments, or detergents). Skin reaction in sensitized or allergic skin belongs to a different category: in this case, the skin that has been sensitized to a specific allergen (sometimes several) and re-exposure to this allergen therefore causes an eczematous reaction (in rare cases contact urticaria or protein contact dermatitis) (Table 1). Other inflammatory dermatoses may be accompanied by similar subjective symptoms but their clinical pictures, as well as histological features, allow specific dermatological diagnose.

**Table 1:** Differences among sensitive, irritated and sensitized/allergic skin.

**Skin type**	**Irritated skin**	**Sensitive skin**	**Allergic skin**
Mechanism	Chemical reaction (irritation)	Neuropathic disorder	Immunological sensitization (allergy)
Triggering factors	Physical, chemical	Physical, chemical	Allergens
Clinical aspects	Caustic dermatitis Irritative dermatitis	Unpleasant sensations and/or erythema	Eczema Contact urticaria Protein contact dermatitis
Main unpleasant sensation	Pain	Paresthesia	Itch
Area	Area of contact	Area of contact and beyond	Area of contact and beyond
Affected individuals	All	Reactive	Allergic

## Assessment and Diagnosis

Sensitive skin is usually diagnosed by the report of unpleasant sensations triggered by some factors (see above) but several sensory testing methods, including stinging tests with either lactic acid or capsaicin and, more rarely, dimethylsulfoxide, can supported the assessment (6, 9, 10). However, the most reliable method to diagnose sensitive skin is based on patient-reported outcomes.

Physical tests such as the stinging test (topical application of a 10% aqueous lactic acid solution on the nasolabial fold compared to the application of physiological saline on the contralateral side) (4) or the thermal sensitivity test (including the capsaicin test) (13) are useful for monitoring the evolution of sensitive skin. However, they are not helpful in diagnosing sensitive skin because they reflect only a limited aspect of cutaneous responsiveness, which is more or less restricted to reactivity to the applied products (14). Nevertheless, the stinging test is widely used, and scores have been defined: the tingling sensation is scored from 0 to 3, and the scores obtained are evaluated and summed 3 and 5 min after application. The sodium lauryl sulfate test is not relevant in sensitive skin because it does not discriminate between sensitive and irritated skin.

The Sensitive Scale is a scale with 10 items (15). The maximum possible score is 100, and the mean score is 44. Nowadays, it is the more frequently used. A specific questionnaire to assess scalp sensitivity, designated the 3S Questionnaire, has also been validated (16). A final score (out of 20) is obtained by multiplying the score severity of abnormal sensations by the total number of sensations. These scales for the self-assessment of sensitive skin are useful tools for self-diagnosis and the evaluation of both the severity of skin sensitivity and the efficacy of treatment. A questionnaire has also been developed to evaluate the burden of sensitive skin; this instrument is known as the Burden of Sensitive Skin (BoSS) questionnaire (17). The BoSS is used to evaluate the severity and evolution of sensitive skin.

Differential diagnosis of sensitive skin seems very easy if the clinician strictly adheres to the definition (7). In practice, however, it might be difficult to distinguish sensitive skin from sensory symptoms related to skin diseases (18), neuropathies (19, 20), or an increase in symptoms due to anxio-depressive or even psychogenic causes (21–23).

The evolution of sensitive skin is chronic, with great variability over time. Thus, sensitive skin undergoes outbreaks followed by remissions. These outbreaks can be affected and aggravated by the menstrual cycle, stress, or seasonal variations (more common in summer). Sensitive skin has an impact on quality of life, at least in its psychological dimension.

## Epidemiological Data

Following the first epidemiological study of sensitive skin was published on a United Kingdom cohort in 2001 (24), other studies have been conducted in many countries throughout the world, including Belgium, France, Germany, Greece, Italy, Portugal, Spain, Switzerland, the United States, Brazil, Japan, Russia, Korea, and India (25). Similar methodologies based on surveys of cohorts aged 15 years and older in accordance with quota sampling, were used in all these studies. The relevant collected information included gender, age, occupation of the head of the household, type of geographical area (rural vs. urban), and region. The relatively low non-response rate to the question “Do you have sensitive skin?” in all countries (<1.5%, except in Russia and Brazil, for which the non-response rate was nearly 10%) suggests that the term “sensitive skin” is known by many individuals. The global prevalence of “sensitive skin” is ~50%, with a great degree of variations among some countries (25). These variations could be explained by genetic or environmental factors but are more likely explained by socio-linguistic factors or cosmetic habits. A comparison of four studies in the USA suggests that the frequency of sensitive skin might increase from 50 to 85% (26) over time, while comparisons of studies on the French population show a smaller increase (27).

Women are more frequently affected than men (60 vs. 40%, respectively). There is a debate on whether the frequency increases with age or decreases in the adult population as well as how common sensitive skin is among children. Variations by ethnicity have been discussed but have not been demonstrated conclusively, which makes sense given the lack of a scientific definition of ethnicities. On the other hand, there are evidenced variations according to phototype and the frequency of sensitive skin, which is increased in people with clear phototypes (12).

Sensitive skin is mainly reported on the face but it can be found in all locations, particularly the hands, scalp, neck and genital area (25, 28, 29). For the scalp (30), the symptomatology is slightly different because erythema is rare (and difficult to diagnose clinically). Pruritus plus tingling is more common than burning plus tingling on the face. When describing the severity of sensitivity, most responses indicated consistency of the perception of sensitive skin on facial and body skin and symptoms in general. However, the pattern was slightly different when describing the skin in the genital area since subjects tended to describe their genital skin as less sensitive than their skin in general (25).

## Pathophysiology

Recently, the IFSI special interest group on sensitive skin provided a position paper on the pathophysiology and management of sensitive skin (31). A multifactorial origin was admitted after discussion of many putative mechanisms. A neurosensory dysfunction in the skin is one of the main pathological mechanisms of sensitive skin (31). Given the high incidence rates it appears problematic to assign a definitive pathophysiological mechanism to sensitive skin (32). However, a growing body of data supports the hypothesis that sensitive skin is a neuropathic disorder (32, 33).

An immunohistochemical study that tested all pathophysiological hypotheses for sensitive skin provided evidences only for the neuronal hypothesis (34): the intra-epidermal nerve fiber density (IENFD) was significantly decreased in patients with sensitive skin, indicating that the Aδ or C fiber population was altered. Furthermore, CGRP immunostaining revealed that the CGRP-immunoreactive nerve fiber density was also reduced (34). A study, involving 70 women with sensitive skin, found that 20% of the patients exhibited characteristics of neuropathic pain based on an evaluation using the DN-4 (*Douleur Neuropathique*-4) questionnaire (35). A recent case-control study on 42 patients showed that both the DN-4 and Neuropathic Pain Symptom Inventory (NPSI) scores were significantly increased in patients with sensitive skin compared with the control group (36), which can be partially explained by some overlapping questions. Using quantitative sensory testing (QST), this study revealed a significant decrease in the heat-pain threshold in the sensitive skin group vs. the control group (36), providing further evidence for the hypothesis that sensitive skin is associated with an alteration of C fibers (37–39). Notably, no difference was found in the vibration detection threshold or the cold detection threshold, which implies the absence of damage to other skin fibers, such as Aβ and Aδ fibers (37–39).

These findings are similar to the criteria for small-fiber neuropathies (SFNs) (20, 40). Patients are considered to exhibit SFN when at least two of the following abnormal results are found:

(1) clinical signs of small-fiber impairment (pinprick and thermal sensory loss or allodynia or hyperalgesia, or any combination of the three) for whichever distribution is consistent with peripheral neuropathy (i.e., length-dependent or non-length-dependent neuropathy);
(2) abnormal warm or cold threshold or both as assessed using quantitative sensory testing (QST); and
(3) reduced IENFD (40).

Hence, the demonstration of a neuropathic component of pain using questionnaires (28, 36) and, above all, the demonstration of a decrease in intra-epidermal density in nerve endings (34) and QST perception abnormalities (36) allow us to consider sensitive skin as a minor equivalent of SFNs, with alterations in cutaneous small nerve fibers, especially in unmyelinated C fibers. These alterations, inducing neuropathic pain and a decrease in heat-pain threshold detection, suggest the hyper-reactivity of nerve endings (41).

Sensitive skin syndrome is frequently associated with irritable bowel syndrome (42) or sensitive eyes (43), which should be related to SFN. Nonetheless, central sensitization to pain and itch may also occur in sensitive skin, sensitive eyes and irritable bowel syndrome (44). The analysis of cerebral responses to cutaneous provocation tests in self-perceived sensitive and non-sensitive skin subjects using functional magnetic resonance imaging (fMRI) (45) showed that cerebral activity was significantly increased in the sensitive skin group. In sensitive skin, activity extended only into the ipsilateral primary sensorimotor cortex and the bilateral peri-insular secondary somatosensory area (45). These findings suggest that, compared with control subjects, subjects with self-perceived sensitive skin exhibit specific cerebral activation during skin irritation tests.

Non-neuronal mechanisms of sensitive skin have been discussed previously. The vascular hypothesis, is still under discussion (14), but erythema is not constant, and vascular modifications are probably consequences of neurogenic activation. Further studies are needed to clarify this aspect. Initially, sensitive skin was associated with the contact of the deep layers of the epidermis with exogenous factors with which they should not have been in contact (based on abnormalities of the epidermal barrier) and thus occurred in clinically unaffected dry skin. This relationship is not confirmed because patients with sensitive skin may have dry, mixed, oily or otherwise normal skin (44, 46). A systematic review of the literature showed that the levels of epidermal pH, sebum production and skin hydration were inconsistent (14). The involvement of keratinocytes remains plausible because keratinocytes express receptors of the transient receptor potential (TRP) family, like TRPV1, or TRPV4.

Two transcriptomic studies have been performed to compare skin samples of patients with sensitive skin and controls using DNA microarray (47) or RNA sequencing (48). Although the small sample size could make the results debatable, the authors showed the involvement of innervation and Merkel cells in the pathophysiology of sensitive skin (49). They also suggested keratinocytic involvement and a putative role of innate immunity (49) or adiponectin deficiency in sensitive skin (47).

Although only reported in mice until now, the recently discovered (50) specialized cutaneous Schwann cells with extensive processes forming a mesh-like network in the subepidermal border of the skin that conveys noxious thermal to the nerve endings might be also involved in the occurrence of sensitive skin syndrome (41). Schwann cells in the skin can activate pain responses (51).

**Figure 1:**
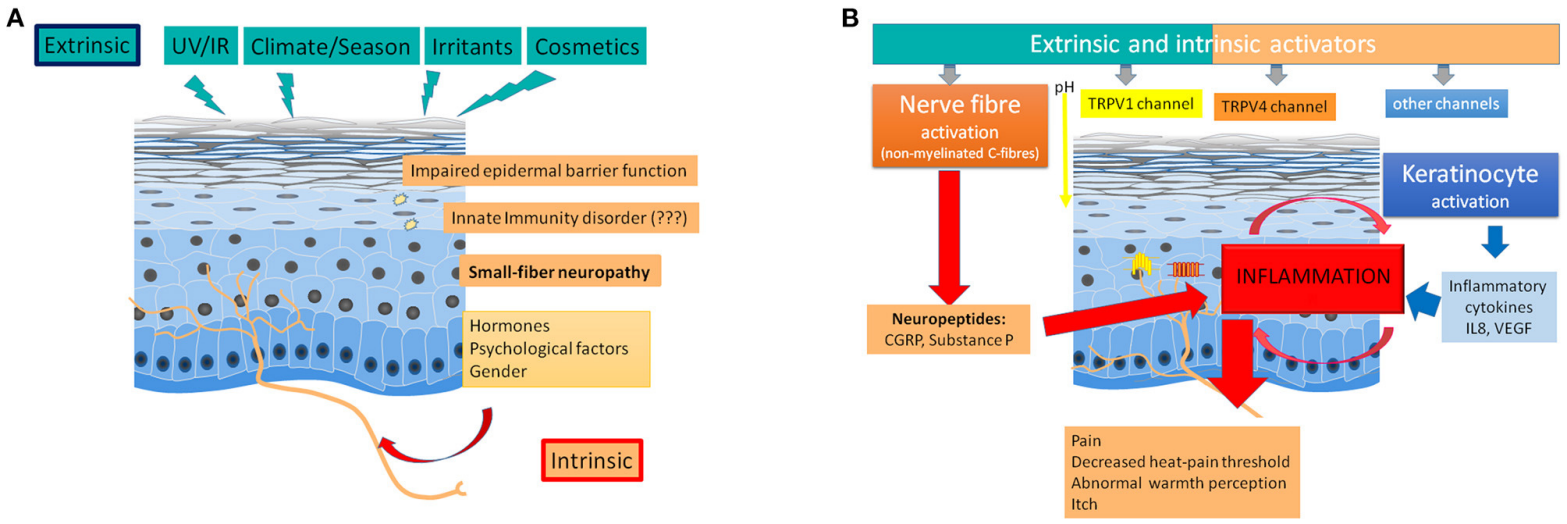
Pathophysiology of sensitive skin. **(A)** Role of extrinsic and intrinsic factors. Extrinsic factors (like radiations, climate, irritants, or cosmetics) as well as intrinsic factors (like, gender, hormones, or psychological factors) can trigger a small-fiber neuropathy, which is frequently associated with an impaired epidermal barrier function. An innate immunity disorder remains discussed. **(B)** Induction of inflammation and abnormal perceptions by nerve fiber (and keratinocyte) activation. Extrinsic and intrinsic factors induce an inflammation after an excessive nerve fiber activation (activation of TRPV1, TRPV4, and other channels followed by the release of neuropeptides in the skin) and a keratinocyte activation (inducing the release of cytokines).

In summary (Figure 1), sensitive skin appears as a disorder of cutaneous small nerve fibers, such as unmyelinated C fibers that mediate pain, itch and warmth (22). These fibers are equipped with sensory neuroreceptors, such as endothelin and TRP channels. These receptors are also expressed by keratinocytes. TRP channels, which were originally described as “polymodal cellular sensors” that can be activated by various physical, chemical and thermal stimuli, are now considered “promiscuous pleiotropic molecules” because the “afferent” functions can be supplemented by “effector” roles (52). The activation of these receptors induces the release of neuropeptides, such as substance P or CGRP, that can cause inflammation, which is termed cutaneous neurogenic inflammation (CNI) (53). Cellular interactions induce the self-maintenance of CNI, which can promote a vicious cycle. Certain G protein-coupled receptors (GPCRs) play a prominent role in these cellular interactions and contribute to self-maintenance. Protease-activated receptors 2 and 4 (PAR-2 and PAR-4, respectively) and Mas-related G protein-coupled receptors (Mrgprs) have been implicated in the synthesis and release of neuropeptides, proteases and soluble mediators from most cutaneous cell types (41). In patients with sensitive skin, this uncontrolled inflammation may be favored by an adiponectin deficiency, with mechanisms that remain poorly understood (47).

## Role of the Environment

Contrary to other SFNs, no relationship with internal disorders is known, but the role of environmental factors is probable (33). Sensitive skin is commonly reported to be induced by various environmental factors, including UV light, cold, heat, cosmetic ingredients and air pollution (22). The hyper-activation of TRP channels (54) represents the most likely mechanism underlying sensitive skin (22). Nonetheless, there is a need for further studies with agonists and antagonists in subjects with sensitive skin and controls to confirm this hypothesis. TRPV1 is activated by capsaicin, H^+^ions, phorbol esters and heat, whereas TRPV3 mediates reactions to warm temperatures and camphor (54). TRPV4 can be activated by heat, mechanical and hypo-osmotic stress as well as UV (54, 55). Both cold and menthol can activate TRPM8, whereas TRPA1 mediates the responses to stimulation by cold, menthol, wasabi and mustard (54). Sensitive skin is related to a decrease in the cutaneous tolerance threshold, which is not linked to allergic or immunological mechanisms, can be observed. Sensitive skin is linked to abnormalities of the cutaneous nervous system, which becomes hyper-reactive. This hyper-reactivity can be modulated by multiple environmental factors.

The evolution of sensitive skin is affected by seasons since it occurs most frequently and more intensely in summer (12), which suggests a role of UV radiation and heat. Likewise, sensitive skin is affected by hormones, since variation can be seen over the menstrual cycle (56).

The role of environmental factors on sensitive skin has been mainly explored through patient interviews. Recently, a literature review with a meta-analysis hierarchized the role of environmental factors in sensitive skin (57). Thirteen studies were included representing a total of 20,486 subjects, who were recruited by random sampling, opportunity sampling or stratified sampling. Questionnaires about triggering factors for sensitive skin were completed through face-to-face meetings, telephone interviews or web surveys. Subjects were classified into “sensitive skin” and “no sensitive skin” groups, and the trigger factors (not aggravating factors) were searched based on different questions (for example, “Is your facial skin easily irritated by …?” or “Do you suffer from burning, prickling or irritation in the presence of …?”). According to the patient-reported outcomes, sensitive skin could be triggered by several factors. The odds ratios of this meta-analysis are presented in Table 2. The most important triggering factor was cosmetics: OR 7.12 (3.98–12.72). Other triggering factors included physical factors (variations in temperature, cold, heat, wind, sun, air conditioning, humid air, and dry air) as well as physico-chemical (water and pollution) and psychological (emotions) (57) factors. Notably, there has been only one exposure study that showed a role of hair dyes as triggering factors for sensitive skin (58). Because the probable role of environmental factors in sensitive skin has mainly been assessed from the patients' point of view, we need further studies to prove the involvement of these factors (e.g., prospective studies with standardized exposure settings).

**Table 2:** Triggering factors of sensitive skin suggested by interviewees in surveys.

**Factor**	**Odds ratio**	**(95% CI)**
Cosmetics	7.12	3.98–12.72
Humid air	3.83	2.48–5.91
Air conditioning	3.60	2.11–6.14
Temperature variation	3.53	2.69–4.63
Heat	3.50	2.56–4.77
Water	3.46	2.82–4.25
Pollution	3.18	2.37–4.27
Dry air	3.04	2.22–4.16
Cold	2.73	1.94–3.84
Wind	2.33	1.69–3.22
Sun	1.81	1.61–2.04
Emotions	1.77	1.44–2.17

*Comparison between people with sensitive skin and healthy subjects. The results are from a meta-analysis (57)*.

## Management

Sensitive skin syndrome needs to be taken into consideration because it can alter the quality of life, especially in women, as measured by either specific scales, such as the DLQI (15) and BoSS (17) questionnaires, or a generic scale, such as SF-12 (12). These alterations have been correlated with the clinical severity of sensitive skin (12, 15, 17). A study using the SF-12 showed that the mental component of quality of life, but not the physical component, was significantly altered (12). Depression or depressive symptoms have not yet been associated with sensitive skin, but subjects who suffer from facial flushing have a significantly higher incidence of depressive symptoms than other subjects (12).

On the basis of an attentive literature review followed by expert meetings, the IFSI special interest group on sensitive skin provides very cautious recommendations (31):

1- The avoidance of possible triggering factors and the use of well-tolerated cosmetics, especially those containing inhibitors of unpleasant sensations, might be suggested for patients with sensitive skin.
2- There is no clinical trial supporting the use of topical or systemic drugs in sensitive skin.
3- There is no study providing data to reach a consensus on the management of sensitive skin.
4- The treatment of sensitive skin can involve the use of well-tolerated cosmetics or cosmetics with effects on cutaneous nervous inflammation, especially on TRPV1 (59, 60). It is therefore necessary to limit their use or use of high tolerance products, that is, products containing few or no preservatives and surfactants. Cosmetics and toiletries seem to be the main triggers for sensitive skin and are probably the main factor sustaining this phenomenon. Since only the exposure study showed an effect of hair dyes on sensitive scalp and facial sensitive skin (58, 61), hair dyes should be avoided, particularly those containing ammonia.
5- Unfortunately, the large majority of commercial products for sensitive skins have no support for their allegations. To our knowledge, there is no study on the use of drugs such as gabapentin, pregabalin or duloxetine.

## Conclusions

Although patients with sensitive skin usually do not meet doctors specifically for this condition, neurologists and other specialists of pain management probably meet them quite often because sensitive skin syndrome is so common. In addition, neurological questioning about sensory signs is a good way to detect them. Therefore, it is useful to be aware of this condition and to avoid considering these outpatients as presenting with functional disorders. If the management is rather the responsibility of dermatologists, it would be interesting to better understand these low-noise SFNs and to follow their evolution toward more serious SFNs. A recent study demonstrated that skin cells (keratinocytes and fibroblasts) from patients with usual small-fiber neuropathies can induce nociceptor degeneration and sensitization (62), suggesting a continuum between sensitive skins and usual small-fiber neuropathies. Indeed, due to the high frequency of sensitive skin syndrome, it is difficult to consider sensitive skins as SFNs in the full sense of this term. This is why we proposed the term “low-noise SFN”.

## Author Contributions

LM and EB gathered all the parts and reviewed the whole article. All authors wrote a part and the review. All authors contributed to the article and approved the submitted version.

## Conflict of Interest

The authors declare that the research was conducted in the absence of any commercial or financial relationships that could be construed as a potential conflict of interest.

## Publisher's Note

All claims expressed in this article are solely those of the authors and do not necessarily represent those of their affiliated organizations, or those of the publisher, the editors and the reviewers. Any product that may be evaluated in this article, or claim that may be made by its manufacturer, is not guaranteed or endorsed by the publisher.

## References

[1] Misery L Brenaut E Le Garrec R Abasq C Genestet S Marcorelles P. Neuropathic pruritus. Nat Rev Neurol. (2014) 10:408–16. 10.1038/nrneurol.2014.99 24912513

[2] Honari G Andersen RM Maibach HI. Sensitive Skin Syndrome. 2nd ed. Boca Raton, FL: CRC Press (2017).

[3] Bernstein ET. Cleansing of sensitive skin; with determination of the pH of the skin following use of soap and a soap substitute. J Invest Dermatol. (1947) 9:5–9. 20262902

[4] Frosch PJ Kligman AM. A method of apraising the stinging capacity of topically applied substances. J Soc Cosmet Chem. (1977) 28:197–209.

[5] Thiers H Peau sensible. In: Thiers H, editors. Les Cosmétiques. 2ème éd. Paris: Masson (1986). p. 266–8.

[6] Berardesca E Fluhr JW Maibach HI. Sensitive Skin Syndrome. New York, NY: Taylor & Francis (2006).

[7] Misery L Ständer S Szepietowski JC Reich A Wallengren J Evers AW. Definition of sensitive skin: an expert position paper from the special interest group on sensitive skin of the international forum for the study of Itch. Acta Derm Venereol. (2017) 97:4–6. 10.2340/00015555-2397 26939643

[8] Guerra-Tapiaa A Serra-Baldrich E Prieto Cabezas L González-Guerraa E López-Estebaranz JL. Diagnóstico y tratamiento del síndrome de piel sensible: un algoritmo para la práctica clínica habitual. Actas Dermosifiliogr. (2019) 110:800–8. 10.1016/j.ad.2018.10.021 31146882

[9] Misery L. Sensitive skin. Expert Rev Dermatol. (2013) 8:631–7. 10.1586/17469872.2013.856688

[10] Misery L Loser K Ständer S. Sensitive skin. J Eur Acad Dermatol Venereol. (2016) 30(Suppl. 1):2–8. 10.1111/jdv.13532 26805416

[11] Kerscher M. Prinzipien der Behandlung und Schutz für empfindliche Haut. Hautarzt. (2011) 62:906–13. 10.1007/s00105-011-2210-1 22160226

[12] Misery L Myon E Martin N Consoli S Boussetta S Nocera T. Sensitive skin: psychological effects and seasonal changes. J Eur Acad Dermatol Venereol. (2007) 21:620–8. 10.1111/j.1468-3083.2006.02027.x 17447975

[13] Jourdain R Bastien P de Lacharriere O Rubinstenn G. Detection thresholds of capsaicin: a new test to assess facial skin neurosensitivity. J Cosmet Sci. (2005) 56:153–66. 16116520

[14] Richters R Falcone D Uzunbajakava N Verkruysse W van Erp P Van de Kerkhof PC. What is sensitive skin? A systematic literature review of objective measurements. Skin Pharmacol Physiol. (2015) 28:75–83. 10.1159/000363149 25322670

[15] Misery L Jean-Decoster C M co S Georgescu V Sibaud V. A new ten-item questionnaire for assessing sensitive skin: the sensitive scale-10. Acta Derm Venereol. (2014) 94:635oli 10.2340/00015555-1870 24710717

[16] Misery L Rahhali N Ambonati M Black D Saint-Martory C Schmitt AM. Evaluation of sensitive scalp severity and symptomatology by using a new score. J Eur Acad Dermatol Venereol. (2011) 25:1295–8. 10.1111/j.1468-3083.2010.03968.x 21241375

[17] Misery L Jourdan E Abadie S Ezzedine K Brenaut E Huet F. Development and validation of a new tool to assess the Burden of Sensitive Skin (BoSS). J Eur Acad Dermatol Venereol. (2018) 32:2217–23. 10.1111/jdv.15186 30022546

[18] Misery L Ständer S. Pruritus. London: Springer (2017).

[19] Oaklander AL. Common neuropathic itch syndromes. Acta Derm Venereol. (2012) 92:118–25. 10.2340/00015555-1318 22307048

[20] Misery L Bodery C Genestet S Zagnoli F Marcorelles P. Small-fibre neuropathies and skin: news and perspectives for dermatologists. Eur J Dermatol. (2014) 24:147. 10.1684/ejd.2013.2189 24509343

[21] Harth W Hermes B Niemeier V Gieler U. Clinical pictures and classification of somatoform disorders in dermatology. Eur J Dermatol. (2006) 16:607–14. 17229599

[22] Misery L. Neuropsychiatric factors in sensitive skin. Clin Dermatol. (2017) 35:281–4. 10.1016/j.clindermatol.2017.01.011 28511825

[23] Misery L Dutray S Chastaing M Schollhammer M Consoli SG Consoli SM. Psychogenic itch. Transl Psychiatry. (2018) 8:52. 10.1038/s41398-018-0097-7 29491364 PMC5830411

[24] Willis CM Shaw S de Lacharriryc O Baverel M Reiche L Jourdain R. Sensitive skin: an epidemiological study. Br J Dermatol. (2001) 145:258–63. 10.1046/j.1365-2133.2001.04343.x 11531788

[25] Farage MA. The prevalence of sensitive skin. Front Med. (2019) 6:98. 10.3389/fmed.2019.00098 31157225 PMC6533878

[26] Farage MA Miller KW Wippel AM Berardesca E Misery L Maibach H. Sensitive skin in the united states: survey of regional differences. Family Med Medical Sci Res. (2013) 2:3.

[27] Misery L Ezzedine K Corgibet F Dupin N Sei JF Philippe C. Sex- and age-adjusted prevalence estimates of skin types and unpleasant skin sensations and their consequences on the quality of life: results from a study of a large representative sample of the French population. Br J Dermatol. (2019) 180:1549–50. 10.1111/bjd.17467 30500070

[28] Saint-Martory C Roguedas-Contios AM Sibaud V Degouy A Schmitt AM Misery L. Sensitive skin is not limited to the face. Br J Dermatol. (2008) 158:130–3. 10.1111/j.1365-2133.2007.08280.x 17986305

[29] Farage MA. Sensitive skin in the genital area. Front Med. (2019) 6:96. 10.3389/fmed.2019.00096 31157224 PMC6529533

[30] Misery L Sibaud V Ambronati M Macy G Boussetta S Taieb C. Sensitive scalp: does this condition exist? An epidemiological study. Contact Dermatitis. (2008) 58:234–8. 10.1111/j.1600-0536.2007.01288.x 18353032

[31] Misery L Weisshaar E Brenaut E Evers AWM Huet F Ständer S. Pathophysiology and management of sensitive skin: position paper from the Special interest Group on sensitive skin of the International Forum for the Study of Itch (IFSI). J Eur Acad Dermatol Venereol. (2020) 34:222–9. 10.1111/jdv.16000 31660659

[32] Schmelz M. Itch processing in the skin. Front Med. (2019) 6:167. 10.3389/fmed.2019.00167 31380380 PMC6659104

[33] Huet F Misery L. Sensitive skin is a neuropathic disorder. Exp Dermatol. (2019) 28:1470–3. 10.1111/exd.13991 31242328

[34] Buhé V Vié K Guéré C Natalizio A Lhéritier C Le Gall-Ianotto C. Pathophysiological study of sensitive skin. Acta Derm Venereol. (2016) 96:314–8. 10.2340/00015555-2235 26337000

[35] Saint-Martory C Sibaud V Theunis J Mengeaud V Lauzé C Schmitt AM. Arguments for neuropathic pain in sensitive skin. Br J Dermatol. (2015) 172:1120–1. 10.1111/bjd.13466 25311408

[36] Huet F Dion A Batardière A Nedelec AS Le Caër F Bourgeois P. Sensitive skin can be small fibre neuropathy: results from a case-control quantitative sensory testing study. Br J Dermatol. (2018) 179:1157–62. 10.1111/bjd.17082 30113701

[37] Backonja MM Attal N Baron R Bouhassira D Drangholt M Dyck PJ. Value of quantitative sensory testing in neurological and pain disorders: NeuPSIG consensus. Pain. (2013) 154:1807–19. 10.1016/j.pain.2013.05.047 23742795

[38] Backonja MM Lauria G. Taking a peek into pain, from skin to brain with ENFD and QST. Pain. (2010) 151:559–60. 10.1016/j.pain.2010.09.016 20937548

[39] Rolke R Magerl W Campbell KA Schalber C Caspari S Birklein F. Quantitative sensory testing: a comprehensive protocol for clinical trials. Eur J Pain. (2006) 10:77–88. 10.1016/j.ejpain.2005.02.003 16291301

[40] Devigili G Tugnoli V Penza P Camozzi F Lombardi R Melli G. The diagnostic criteria for small fibre neuropathy: from symptoms to neuropathology. Brain. (2008) 131:1912–25. 10.1093/brain/awn093 18524793 PMC2442424

[41] Misery L. Sensitive skins may be neuropathic disorders: lessons from studies on skin and other organs. Cosmetics. (2021) 8:14. 10.3390/cosmetics8010014

[42] Misery L Duboc H Coffin B Brenaut E Huet F Taieb C. Association between two painful and poorly understood conditions: irritable bowel and sensitive skin syndromes. Eur J Pain. (2019) 23:160mes 10.1002/ejp.1296 30076662

[43] Misery L Cochener B Brenaut ES ena S Taieb C. Association of sensitive skin with sensitive corneas and sensitive eyelids. J Eur Acad Dermatol Venereol. (2019) 33:1358–358 10.1111/jdv.15595 30903713

[44] Misery L Boussetta S Nocera T Perez-Cullell N Taieb C. Sensitive skin in Europe. J Eur Acad Dermatol Venereol. (2009) 23:376–81. 10.1111/j.1468-3083.2008.03037.x 19335729

[45] Querleux B Dauchot K Jourdain R Bastien P Bittoun J Anton JL. Neural basis of sensitive skin: an fMRI study. Skin Res Technol. (2008) 14:454–61. 10.1111/j.1600-0846.2008.00312.x 18937781

[46] Misery L Jourdan E Huet F Brenaut E Cadars B Virassamynajd S. Sensitive skin in france: a study on prevalence, relationship with age and skin type and impact on quality of life. J Eur Acad Dermatol Venereol. (2018) 32:791ols 10.1111/jdv.14837 29397030

[47] Kim EJ Lee DH Kim YK Eun HC Chung JH. Adiponectin deficiency contributes to sensitivity in human skin. J Invest Dermatol. (2015) 135:2331–234. 10.1038/jid.2015.150 25880703

[48] Yang L Lyu L Wu W Lei D Tu Y Xu D. Genome-wide identification of long non-coding RNA and mRNA profiling using RNA sequencing in subjects with sensitive skin. Oncotarget. (2017) 8:114894–910. 10.18632/oncotarget.23147 29383128 PMC5777740

[49] Bataille A Le Gall-Ianotto C Genin E Misery L. Sensitive skin: lessons from transcriptomic studies. Front Med. (2019) 6:115. 10.3389/fmed.2019.00115 31192213 PMC6546803

[50] Abdo H Calvo-Enrique L Martinez Lopez J Song J Zhang MD Usoskin D. Specialized cutaneous schwann cells initiate pain sensation. Science. (2019) 365:695–9. 10.1126/science.aax6452 31416963

[51] Doan RA. Monk KR. Glia in the skin activate pain responses. Science. (2019) 365:641–2. 10.1126/science.aay6144 31416950

[52] Tóth BI Oláh A Szöllosi AG Bíró T. TRP channels in the skin. Br J Pharmacol. (2014) 171:2568–81. 10.1111/bph.12569 24372189 PMC4009000

[53] Gouin O Lebonvallet N L'Herondelle K Le Gall-Ianotto C Buhé V Plée-Gautier E. Self-maintenance of neurogenic inflammation contributes to a vicious cycle in skin. Exp Dermatol. (2015) 24:723–6. 10.1111/exd.12798 26178975

[54] Caterina MJ Pang Z. TRP channels in skin biology and pathophysiology. Pharmaceuticals. (2016) 9:E77. 10.3390/ph9040077 27983625 PMC5198052

[55] Moore C Cevikbas F Pasolli HA Chen Y Kong W Kempkes C. UVB radiation generates sunburn pain and affects skin by activating epidermal TRPV4 ion channels and triggering endothelin-1 signaling. Proc Natl Acad Sci USA. (2013) 110:E3225–334. 10.1073/pnas.1312933110 23929777 PMC3752269

[56] Falcone D Richters R Uzunbajakava N Van Erp P Van de Kerkhof PC. Sensitive skin and the influence of female hormone fluctuations: results from a cross-sectional digital survey in the Dutch population. Eur J Dermatol. (2017) 27:42–8. 10.1684/ejd.2016.2913 27873738

[57] Brenaut E Barnetche T Le-Gall Ianotto C Roudot AC Misery L Ficheux AS. Role of the environment in sensitive skin from the worldwide patient's point of view: a literature review and meta-analysis. J Eur Acad Dermatol Venereol. (2020) 34:230–8. 10.1111/jdv.15985 31571336

[58] Bernard A Houssin A Ficheux AS Wesolek N Nedelec AS Bourgeois P. Consumption of hair dye products by the French women population: usage pattern and exposure assessment. Food Chem Toxicol. (2016) 88:123–32. 10.1016/j.fct.2016.01.002 26763610

[59] Fauger A Lhoste A Chavagnac-Bonneville M Sayag M Jourdan E Ardiet N. Effects of a new topical combination on sensitive skin. J Cosmet Sci. (2015) 66:79–86. 26454972

[60] Schoelermann AM Jung KA Buck KA Grönniger E Conzelmann S. Comparison of skin calming effects of cosmetic products containing 4-t-butylcyclohexanol or acetyl dipeptide-1 cetyl ester on capsaicin-induced facial stinging in volunteers with sensitive skin. J Eur Acad Dermatol Venereol. (2016) 30(Suppl. S1):18–20. 10.1111/jdv.13530 26805418

[61] Bernard A Ficheux AS Nedelec AS Bourgeois P Hornez N Batardiiss A. Induction of sensitive skin and sensitive scalp by hair dyeing. Int J Eng Res Gen Sci. (2016) 4:5.

[62] Kreß L Hofmann L Klein T Klug K Saffer N Spitzel M. Differential impact of keratinocytes and fibroblasts on nociceptor degeneration and sensitization in small fiber neuropathy. Pain. (2021) 162:1262–72. 10.1097/j.pain.0000000000002122 33196576

